# First in-human quantitative plaque characterization with ultra-high resolution coronary photon-counting CT angiography

**DOI:** 10.3389/fcvm.2022.981012

**Published:** 2022-09-06

**Authors:** Victor Mergen, Matthias Eberhard, Robert Manka, André Euler, Hatem Alkadhi

**Affiliations:** ^1^Institute of Diagnostic and Interventional Radiology, University Hospital Zurich, University of Zurich, Zurich, Switzerland; ^2^Department of Cardiology, University Heart Center, University Hospital Zurich, University of Zurich, Zurich, Switzerland

**Keywords:** coronary computed tomographic angiography (CCTA), coronary artery disease, high risk plaque, ultra-high-resolution CT, photon-counting detector CT (PCD-CT)

## Abstract

**Purpose:**

To assess the effect of ultra-high-resolution coronary CT angiography (CCTA) with photon-counting detector (PCD) CT on quantitative coronary plaque characterization.

**Materials and methods:**

In this IRB-approved study, 22 plaques of 20 patients (7 women; mean age 77 ± 8 years, mean body mass index 26.1 ± 3.6 kg/m^2^) undergoing electrocardiography (ECG)-gated ultra-high-resolution CCTA with PCD-CT were included. Images were reconstructed with a smooth (Bv40) and a sharp (Bv64) vascular kernel, with quantum iterative reconstruction (strength level 4), and using a slice thickness of 0.6, 0.4, and 0.2 mm, respectively (field-of-view 200 mm × 200 mm, matrix size 512 × 512 pixels). Reconstructions with the Bv40 kernel and slice thickness of 0.6 mm served as the reference standard. After identification of a plaque in coronary arteries with a vessel diameter ≥2 mm, plaque composition was determined using a dedicated, semi-automated plaque quantification software. Total plaque, calcified, fibrotic, and lipid-rich plaque components were quantified in all datasets.

**Results:**

Median plaque volume was highest (23.5 mm^3^, interquartiles 17.9–34.3 mm^3^) for reconstructions with the reference standard and lowest for ultra-high-resolution reconstructions with a slice thickness of 0.2 mm and the Bv64 kernel (18.1 mm^3^, interquartiles 14.1–25.8 mm^3^, *p* < 0.001). Reconstructions with the reference standard showed largest calcified (85.1%, interquartiles 76.4–91.1%) and smallest lipid-rich plaque components (0.5%, interquartiles 0.0–1.5%). Smallest calcified plaque components (75.2%, interquartiles 69.9–80.8%) and largest lipid-rich components (6.7%, interquartiles 5.1–8.4%) were found for ultra-high-resolution reconstructions with a slice thickness of 0.2 mm and the Bv64 kernel. At an identical slice thickness, volume of calcified components was always lower, and volume of lipid-rich components was always higher for reconstructions with the Bv64 kernel compared with reconstructions with the Bv40 kernel (all, *p* < 0.001).

**Conclusion:**

This patient study indicates significant differences of ultra-high-resolution scanning with PCD-CT on quantitative coronary plaque characterization. Reduced blooming artifacts may allow improved visualization of fibrotic and lipid-rich plaque components with the ultra-high-resolution mode of PCD-CT.

## Introduction

Coronary CT angiography (CCTA) is the primary imaging modality for the non-invasive anatomical assessment of coronary artery disease (1–5). CCTA provides an excellent visualization of atherosclerotic plaques and enables the characterization of certain coronary plaque types (2, 6–10). While macrocalcified plaques are considered to be relatively stable, low attenuation plaques, positive remodeling, the napkin ring sign, and spotty calcifications on CCTA have been associated with plaque instability (6, 7, 11). Moreover, total burden of low-attenuating coronary plaques has been identified as the strongest predictor of adverse coronary events in patients with acute and chronic coronary syndrome (6, 12). Thus, precise plaque characterization and quantification is decisive to accurately identify patients at risk for future cardiovascular events.

Current plaque characterization in clinical routine is often based on a pure, subjective visual assessment or on the measurement of plaque attenuation (6, 7, 13, 14). However, blooming artifacts may lead to an overestimation of the volume of calcified plaques and to difficulties in the delineation of adjacent, noncalcified components. In addition, precise separation of plaque borders to the adventitial layer and to the epicardial adipose tissue may be hampered because of only subtle attenuation differences between these structures (15).

Novel photon-counting detector CT (PCD-CT) offers the possibility to acquire ECG-gated ultra-high-resolution CCTA. The direct conversion process of detected X-ray photons used in PCD-CT optimizes the geometric dose efficiency, enabling a maximum in-plane resolution of 0.11 mm × 0.11 mm and a maximum through-plane resolution of 0.16 mm (16, 17). PCD-CT has been shown to outperform conventional energy-integrating detector CT with regard to the visualization of calcified and noncalcified plaques along with a significant reduction of blooming artifacts (16, 18). Considering ultra-high-resolution CCTA with PCD-CT, reconstruction parameters such as the slice thickness, matrix size, and reconstruction kernel need to be carefully chosen as they have a major influence on visualization of the coronary arteries and hence, on atherosclerotic wall changes (16).

The aim of this study was to assess the effect of reconstruction parameters on quantitative coronary plaque characterization in patients undergoing ultra-high-resolution CCTA with PCD-CT. Our hypothesis was that the high spatial resolution in combination with the optimal reconstruction kernel would enable a better visualization of coronary plaques with less blooming and improved delineation of non-calcified plaque components compared with standard reconstructions.

## Materials and methods

### Patients

Consecutive patients undergoing ultra-high-resolution CCTA prior to transcatheter aortic valve replacement between February and May 2022 were screened for study inclusion. Patients were included when atherosclerotic plaques in proximal coronary arteries with a vessel diameter equal to or larger than 2 mm were present.

This retrospective single-center study was approved by the local ethics committee and was performed in compliance with the declaration of Helsinki. All patients are part of a nation-wide TAVR registry (clinicalTrials.gov, Identifier: NCT01368250) and provided written informed consent.

### CT data acquisition and image reconstruction

CT was performed on a whole-body, dual-source PCD-CT system (NAEOTOM Alpha; version syngo CT VA50; Siemens Healthcare GmbH; Forchheim, Germany) equipped with two cadmium telluride detectors. All patients were pretreated with sublingual nitroglycerin (2.5 mg isosorbide dinitrate). No beta-blockers for heart rate control were administered. Scan acquisition included an ECG-gated unenhanced cardiac scan for calcium scoring followed by an ECG-gated ultra-high-resolution CCTA and a thoracoabdominal CT aortography.

Ultra-high-resolution CCTA was initiated with bolus tracking by placing a region of interest (ROI) in the ascending aorta. Depending on the body weight of the patients, a total volume of 50–70 ml contrast material (Iopromide, Ultravist 370 mg iodine/ml, Bayer Healthcare, Berlin, Germany) was administered with a power injector at a weight-based flow rate of 3.3–4.4 ml/s through an 18-gauge needle placed into an antecubital vein. After contrast material injection, a saline chaser (NaCl 0.9%) of 20 ml was administered applying the same flow rate. Scan start was initiated after the attenuation within the ROI exceeded 140 Hounsfield units (HU) at 90 kV. Ultra-high-resolution CCTA image data was acquired with a retrospective ECG-gated dual-source helical (pitch 0.22–0.28 depending on the heart rate) or a prospective ECG-gated dual-source sequential mode. Scan parameters were as follows: tube voltage 120 kV, collimation 120 × 0.2 mm, tube current-time product at an image quality level of 64 using automated tube current modulation. The constant image quality level ensured similar image quality across different scan modes. Heart rate dependent ECG-pulsing was applied.

After selection of a cardiac phase with no motion artifacts, ultra-high-resolution CCTA images were reconstructed using three different slice thicknesses (0.6, 0.4, and 0.2 mm, respectively) and two kernels with different sharpness levels (smooth Bv40 kernel and sharp Bv64 kernel, respectively). Whole spectrum of detected photons employing a single energy threshold at 20 keV is used to reconstruct the ultra-high-resolution CCTAs. The Bv40 kernel is the default vascular kernel recommended by the manufacturer for CCTA, while the Bv64 was determined as the optimal kernel for the reconstruction of ultra-high-resolution CCTA in a previous study (16). Quantum iterative reconstruction (19) at a strength level of 4 was applied to all reconstructions. Field-of-view and matrix size were set to 200 mm × 200 mm and 512 × 512 pixels, respectively. The reconstruction with a slice thickness of 0.6 mm, increment of 0.3 mm, and using the default Bv40 kernel served as the reference standard for further comparisons.

### Quantitative plaque analysis

**FIGURE 1:**
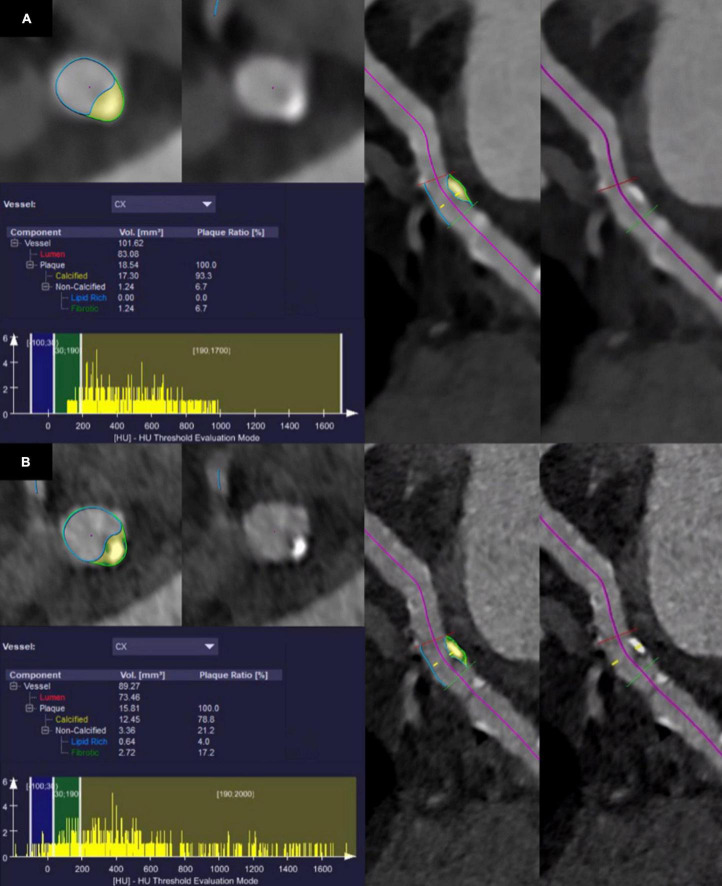
Segmentation and quantitative results of plaque characteristics in the circumflex artery of a 75-year-old male patient who underwent coronary CT angiography with photon-counting detector CT. Note the shift of CT values in the histogram towards lower values using ultra-high-resolution reconstructions with a slice thickness of 0.2 mm and the Bv64 kernel **(B)** compared with reconstructions with a slice thickness of 0.6 mm and the Bv40 kernel **(A)**.

Quantitative evaluation of atherosclerotic plaques was performed using a semiautomatic software (CT Coronary Plaque Analysis, software version 5.0.2, Syngo.via VB60A, Siemens Healthcare GmbH; Forchheim, Germany) (20), previously evaluated by Tesche et al. (21). After automatic centerline extraction of the coronary arteries, one trained reader (V.M., resident with three years of experience in cardiovascular imaging) manually defined the proximal and the distal aspect of the coronary artery segment with atherosclerotic plaque. In the next step, the software automatically detected the outer and inner vessel wall including the atherosclerotic plaque. If the segmentation was inaccurate, manual adjustments were performed by the reader. Finally, plaque constituents were assessed based on their attenuation using preset HU ranges. Non-calcified plaque components were considered as low-attenuating, lipid-rich when showing an attenuation between −100 and 29 HU and as intermediate, fibrotic when showing an attenuation between 30 and 189 HU (22, 23). Plaque components were considered calcified when showing an attenuation above 190 HU. Software output included the total plaque volume, volume and plaque ratio of the calcified, fibrotic, and lipid-rich components as well as a histogram displaying all CT attenuation values. Quantitative results were noted. If more atherosclerotic plaques were present in one coronary artery, only the most proximal plaque was considered. For each plaque, segmentation and characterization was performed on all six reconstructed datasets, carefully selecting identical proximal and distal aspects of the coronary artery segment. Figure 1 provides a representative example of the quantitative plaque analysis process.

### Statistical analysis

Continuous variables were assessed by Shapiro–Wilk tests for normal distribution. Normally distributed variables are presented as mean ± SD, whereas non-normally distributed variables are reported as median and interquartiles. Friedman tests followed by Wilcoxon-tests served to check for differences in non-normally distributed values. The Benjamini–Hochberg procedure was applied to adjust for multiple comparisons. A two-tailed *p*-value < 0.05 was considered to infer statistical significance. Categorical variables are reported as counts and percentages. All statistical analyses were performed using R statistical software (R, version 4.1.1; R Foundation for Statistical Computing, Vienna, Austria^1^).

## Results

Among 25 patients undergoing ultra-high-resolution CCTA with PCD-CT, 3 patients were excluded because of the absence of atherosclerotic plaques in the proximal coronary arteries and 2 patients were excluded because of extensive calcified plaques in the proximal coronary arteries that extended to distal segments. No patients were excluded because of other criteria (e.g., motion artifacts). Finally, a total of 22 plaques from 20 patients were included (7 women, 13 men, mean age 77 ± 8 years, mean body mass index 26.1 ± 3.6 kg/m^2^). Nine scans were acquired with retrospective ECG-gating [mean volume CT dose index (CTDI_vol_) 36.8 ± 6.3 mGy]; 11 scans were acquired with prospective ECG-gating (mean CTDI_vol_ 28.1 ± 6.8 mGy). The average heart rate was 69 ± 15 beats per minute during data acquisition. Patient demographics are shown in Table 1. Eight out of 20 patients were also included in the patient sample of our previous study on image reconstruction parameters of ultra-high-resolution CCTA (16).

**TABLE 1:** Patient demographics.

Characteristic	All patients (*n* = 18)
**Sex**	
Male	13 (65%)
Female	7 (35%)
Age (years)	77 ± 8 (range, 63–90)
Body weight (kg)	75 ± 11 (range, 54–96)
Body mass index (kg/m^2^)	26.1 ± 3.6 (range, 19.6–33.3)
Heart rate during the data acquisition (bpm)	69 ± 15 (range, 52–118)
**Blood pressure (mmHg)**	
Systolic	149 ± 30 (range, 96–206)
Diastolic	81 ± 17 (range, 57–119)
Coronary artery calcium score*	416 (298–917)
**Medical history**	
Diabetes	4/20 (20%)
Dyslipidemia	8/20 (40%)
Arterial hypertension	16/20 (80%)
Smoking history	7/20 (35%)
Chronic obstructive pulmonary disease	3/20 (15%)
Chronic kidney disease	2/20 (10%)

Unless otherwise indicated, data are mean ± SD.

*n*, number of patients; bpm = beats per minute.

*Data is median; data in parentheses are interquartiles.

### Quantitative plaque analysis

**FIGURE 2:**
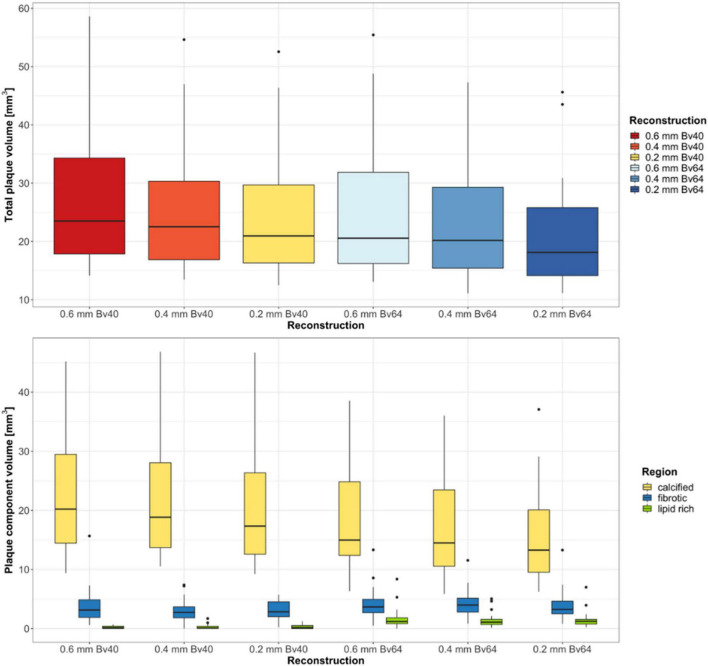
Results of the quantitative plaque analysis. Boxplots at the top show the total plaque volume and boxplots at the bottom depict the volume of the different plaque components determined on reconstructions with three different slice thicknesses and two different kernel strengths. Horizontal lines in the boxes indicate the median, and the top and bottom lines of boxes indicate the first and third quartiles, respectively. Whiskers show lowest and highest values within 1.5 interquartile range of the lower and upper limits, and circles indicate outliers.

Twenty-two plaques in six image datasets each were analyzed. Two plaques were located in the left main artery, eight plaques in the left anterior descending artery, five plaques in the circumflex artery, and seven plaques in the right coronary artery. A summary of the quantitative results is shown in Table 2. Total plaque volume (*p* < 0.001) and volume of calcified (*p* < 0.001), fibrotic (*p* = 0.003), and lipid rich components (*p* < 0.001) significantly differed across all reconstructions (Figure 2). Median total plaque volume was highest for reconstructions with the reference standard (slice thickness 0.6 mm and Bv40 kernel: 23.5 mm^3^, interquartiles 17.9–34.3 mm^3^) and smallest for ultra-high-resolution reconstructions with a slice thickness of 0.2 mm and the Bv64 kernel (18.1 mm^3^, interquartiles 14.1–25.8 mm^3^, *p* < 0.001, *post hoc* Wilcoxon test). Total plaque volume and volume of calcified components were on average 23 ± 6% and 32 ± 7% lower on reconstructions with a slice thickness of 0.2 mm and the Bv64 kernel compared with the reference standard. At identical slice thickness, volume of calcified components was always lower and volume of lipid-rich components was always higher (all, *p* < 0.001, *post hoc* Wilcoxon tests) while volume of fibrotic components was similar (all, *p* > 0.05, *post hoc* Wilcoxon tests) on reconstructions with the Bv64 kernel compared with reconstructions with the Bv40 kernel.

**TABLE 2:** Results of quantitative plaque analysis.

	Total plaque	Calcified component		Fibrotic component		Lipid rich component	
Reconstruction	Volume (mm^3^)	Volume (mm^3^)	Plaque ratio (%)	Volume (mm^3^)	Plaque ratio (%)	Volume (mm^3^)	Plaque ratio (%)
0.6 mm Bv40	23.5 (17.9–34.3)	20.2 (14.4–29.5)	85.1 (76.4–91.1)	3.1 (1.9–4.9)	14.0 (8.5–21.3)	0.2 (0.0–0.4)	0.5 (0.0–1.5)
0.4 mm Bv40	22.5 (16.9–30.3)	18.8 (13.7–28.1)	88.3 (84.6–91.1)	2.7 (1.8–3.7)	10.9 (8.4–15.2)	0.1 (0.0–0.4)	0.5 (0.1–0.9)
0.2 mm Bv40	21.0 (16.3–29.7)	17.3 (12.6–26.4)	84.2 (81.3–89.3)	2.8 (2.0–4.5)	13.6 (9.9–17.4)	0.2 (0.0–0.5)	0.9 (0.0–2.2)
0.6 mm Bv64	20.6 (16.2–31.9)	15.0 (12.4–24.8)	77.5 (66.8–82.9)	3.7 (2.7–5.0)	16.4 (13.0–21.6)	1.2 (0.8–1.8)	5.4 (3.2–10.5)
0.4 mm Bv64	20.2 (15.4–29.3)	14.5 (10.6–23.5)	76.6 (69.9–79.2)	4.0 (2.8–5.1)	17.8 (15.6–23.2)	1.1 (0.7–1.6)	5.2 (3.7–8.3)
0.2 mm Bv64	18.1 (14.1–25.8)	13.3 (9.5–20.1)	75.2 (69.9–80.8)	3.2 (2.5–4.6)	18.6 (15.0–21.0)	1.2 (0.8–1.6)	6.7 (5.1–8.4)

Data are medians, data in parentheses are interquartiles.

Reconstructions with the reference standard yielded the highest ratio of calcified (85.1%, interquartiles 76.4–91.1%) and the lowest ratio of lipid-rich plaque components (0.5%, range 0.0–1.5%). Lowest ratio of calcified plaque components (75.2%, interquartiles 69.9–80.8%) and highest ratio of lipid rich components (6.7%, interquartiles 5.1–8.4%) were found for reconstructions with a slice thickness of 0.2 mm and the Bv64 kernel.

**FIGURE 3:**
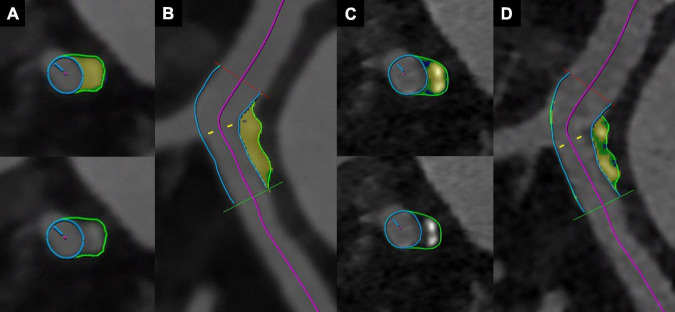
Coronary CT angiography with photon-counting detector CT of an 88-year-old male patient. Images show a mixed plaque reconstructed with a slice thickness of 0.6 mm and the Bv40 kernel **(A,B)**, and with a slice thickness of 0.2 mm and the Bv64 kernel **(C,D)**. Note the improved visualization of non-calcified components (blue color corresponds to lipid rich components, green color to fibrotic components, and yellow color to calcified components) at a slice thickness of 0.2 mm with the Bv64 kernel **(C,D)**.

A representative example of improved plaque visualization along with the differences in the automated segmentation of different plaque components is shown in Figure 3.

## Discussion

CT-based coronary plaque characterization and quantification may help to identify patients at risk for future adverse cardiac events (6, 7, 12, 24, 25). PCD-CT allows for the acquisition of CCTA image data with a high in-plane and through-plane resolution of 0.11 mm × 0.11 mm and 0.16 mm, respectively. In this study, we evaluated the effect of spatial resolution in combination with various reconstruction kernels on quantitative plaque characterization using ultra-high-resolution CCTA. Our results indicate significantly lower total plaque volumes and significantly lower calcified plaque components on ultra-high-resolution reconstructions with a slice thickness of 0.2 mm and using the Bv64 kernel as compared with reference standard reconstructions. Interestingly, the volume of non-calcified plaque (i.e., both fibrotic and lipid-rich) components was highest on ultra-high-resolution reconstructions with the thinnest slice thickness and with the sharp vascular kernel.

The clinical PCD-CT system allows for two acquisition modes for ECG-gated CCTA, i.e., the ultra-high-resolution mode and the spectral (QuantumPlus) mode. The ultra-high-resolution mode used in this study is characterized by a so-far unprecedented spatial resolution for clinical CT when applying a collimation of 120 × 0.2 mm. Detector pixels are read out separately and so-called “T3D” images are reconstructed employing the whole spectrum of absorbed photons and using a single energy threshold at 20 keV(16, 26). Currently, this mode does not allow for collecting additional spectral information. The spectral (QuantumPlus) mode generates spectral information, while the spatial resolution is limited to a collimation of 144 × 0.4 mm (27). CCTA with spectral information enables the computation of virtual noniodine images (PureCalcium), potentially obviating the need for true noncontrast scans (28) and for the reconstruction of images with a virtual, dual energy-based removal of calcified plaques (PureLumen), potentially facilitating coronary artery stenosis assessment (29). The spectral mode enables also the dual energy-based characterization of plaque components (20), which would be a potential advantage compared with the ultra-high-resolution mode. In an experimental study, PCD-CT operated in a multi-energy mode even allowed for the detection and quantification of the macrophage burden within calcified atherosclerotic plaques using gold nanoparticles (30).

Mergen et al. were the first to assess the feasibility of ultra-high-resolution PCD-CCTA in patients and to perform a systematic evaluation of the optimal reconstruction parameters including slice thickness and reconstruction kernel (16). They observed that slice thickness as well as the reconstruction kernel had a major impact on vessel sharpness as well as blooming artifacts. Based on their quantitative and qualitative results, they identified the Bv64 kernel to be the optimal kernel for the reconstruction of ultra-high-resolution CCTA. Findings of this previous study formed the rationale to investigate the influence of slice thickness on plaque characterization applying the Bv40 and the Bv64 kernel.

In our study, total plaque volume and volume of calcified plaque components was smallest on reconstructions with a slice thickness of 0.2 mm and the Bv64 kernel. This suggests that higher spatial resolution results in lower blooming artifacts from calcium. These results are consistent with a study of Si-Mohamed et al. in which image quality and diagnostic confidence of CCTA acquired with conventional CT and with a prototype PCD-CT system were compared (18). These authors observed a significant reduction of blooming artifacts and a considerable improvement in the visualization of noncalcified plaques on CCTA images using PCD-CT with a slice thickness of 0.25 mm.

Subtle plaque characteristics on CCTA such as low attenuation plaque components, positive remodeling, spotty calcifications, and the napkin-ring sign type have been identified as features of high-risk plaques (7, 11, 31). It is important to mention that virtually all calcified plaques have non-calcified plaque components as well (32). This is corroborated by the recent findings of Yin et al. who identified four unstable plaques on histopathology that appeared as purely calcified plaques on CCTA (33). Thus, it must be the aim to reduce blooming artifacts in CCTA for visualization of non-calcified components. In our study, we found the highest volume of lipid-rich plaque components on ultra-high-resolution reconstructions (slice thickness 0.2 mm, Bv64 kernel), whereas smallest were found in the reference standard reconstructions. Thus, ultra-high-resolution CCTA with PCD-CT has the potential to improve patient risk stratification.

It should be noted that all available thresholds for morphometric plaque analysis were established for standard resolution CCTA acquired with conventional energy-integrating detector CT and reconstructed as polychromatic images (22, 23). Although T3D images of PCD-CT emulate polychromatic images of EID-CT (34), thresholds have not yet been validated for PCD-CT.

The following study limitations merit consideration. First, this single-center retrospective study encompassed only a limited number of plaques and patients. Second, differentiation of plaque components was entirely based on quantitative CT attenuation differences and lacks correlation to intravascular ultrasound or intravascular optical coherence tomography. Third, no outcome data was available in our patients. Fourth, all ultra-high-resolution images were reconstructed using QIR at a strength level of 4, and the influence of different QIR strength levels on plaque characterization was not analyzed. Fifth, we did not evaluate the reproducibility of plaque quantification with the software tool used in our study. Finally, patients included in this study had a stable coronary artery disease with a high burden of coronary artery calcifications (median coronary artery calcium score of 456). In a patient sample presenting with acute chest pain or acute coronary syndrome divergent results with a higher ratio of noncalcified plaque components are to be expected.

## Conclusion

In conclusion, ultra-high-resolution CCTA with PCD-CT using ultra-high-resolution reconstructions (slice thickness 0.2 mm) and using a sharp vascular kernel (Bv64) yield smallest calcified and largest non-calcified coronary plaque components, which suggests reduced blooming artifacts and better visualization of non-calcified plaque components, the latter being considered important for individualized risk prediction. Certainly, future studies in a larger patient cohort with comparison of the ultra-high-resolution mode with intravascular ultrasound, intravascular optical coherence tomography, or histopathology are needed. In addition, the potential improvement for optimized risk prediction of this scan mode in comparison with conventional, energy-integrating detector CT should be addressed.

## Data availability statement

The raw data supporting the conclusions of this article will be made available by the authors, without undue reservation.

## Ethics statement

The studies involving human participants were reviewed and approved by the Kantonale Ethikkommission Zürich. The patients/participants provided their written informed consent to participate in this study.

## Author contributions

HA and VM conceived this study design. VM performed data collection, image analysis, statistical analysis, interpreted the data, and prepared manuscript draft. HA supervised this study, interpreted the data, and prepared manuscript draft. ME, RM, and AE prepared manuscript draft. All authors contributed to the article and approved the submitted version.

## Footnotes

1
https://www.R-project.org/
